# Seven Years Follow-Up after Sleeve Gastrectomy in Adolescents

**DOI:** 10.1007/s11695-025-07966-7

**Published:** 2025-06-16

**Authors:** Mohamed Shehata, Wael Abosena, Ahmed Elhaddad, Ashraf El Attar

**Affiliations:** Tanta University, Tanta, Egypt

**Keywords:** Adolescent, Obesity, Sleeve gastrectomy, Long-term outcomes, Weight loss, Comorbidity, Fertility

## Abstract

**Background:**

Adolescent obesity is a growing global health challenge, often accompanied by serious comorbidities. This study evaluates the long-term outcomes of laparoscopic sleeve gastrectomy (LSG) in adolescents, focusing on weight loss, comorbidity resolution, and post-surgical complications over a seven-year period.

**Materials and Methods:**

A retrospective analysis was conducted on 63 adolescents (mean age: 15.4 ± 2.39 years) at Tanta university hospitals, Egypt, who underwent LSG between 2014 and 2015. Outcomes assessed included body mass index (BMI), percent total weight loss (%TWL), percent excess weight loss (%EWL), excess BMI loss (%EBMIL), comorbidity resolution, and gastric volumetry at 1, 3, 5, and 7 years postoperatively.

**Results:**

Mean BMI declined from 48.1 ± 10.55 to 29.3 ± 8.53 kg/m^2^, with %TWL peaking at 32.9% at year 3 and moderating to 29.1% by year 7. Significant reductions were observed in diabetes (34.9% to 11.1%), hypertension (60.3% to 12.7%), obstructive sleep apnea (39.7% to 6.4%), GERD (25.4% to 4.8%), and dyslipidemia (42.9% to 3.2%) (all p < 0.05). The overall complication rate was low (7.9%), and 14.3% required revisional surgery. Among married participants, 76.9% achieved successful pregnancies.

**Conclusions:**

LSG is a safe and effective long-term intervention for adolescents with severe obesity, yielding sustained weight loss and significant comorbidity remission, with favorable fertility outcomes among married patients. Despite modest weight regain and a limited rate of reoperations, outcomes support LSG as a durable option. Long-term follow-up and individualized care remain essential.

## Introduction

Adolescent obesity poses a major worldwide public health concern, especially due to its strong links to various comorbid conditions such as cardiovascular disease, diabetes mellitus, hypertension, and obstructive sleep apnea [1]. In adolescents, Obesity is classified as a body mass index (BMI) that meets or exceeds the 95 th percentile, as determined by growth charts tailored to age and gender [2]. These complications often persist into adulthood, emphasizing the need for early intervention [3].

For severely obese adolescents, bariatric surgery has become a viable therapeutic approach when conventional methods prove insufficient [4]. While Roux-en-Y gastric bypass (RYGB) was historically the predominant procedure [5], laparoscopic sleeve gastrectomy (LSG) has recently gained prominence as the preferred surgical intervention, mainly due to its lower surgical risk profile [6].

Long-term studies have demonstrated favorable outcomes for LSG in adolescents, including significant resolution of comorbid conditions such as depression, hypertension, gastroesophageal reflux disease (GERD), dyslipidemia, obstructive sleep apnea (OSA), and type 2 diabetes (T2DM) [7, 8]. The procedure’s metabolic benefits extend beyond weight loss, with studies indicating substantial improvements in insulin sensitivity and lipid profiles [9] and overall quality of life [10].

Current literature on adolescent LSG has several limitations, including modest sample sizes [11] and insufficient assessment of psychological outcomes including quality of life, self-perception of body image, and concerns related to body esteem [12]. These gaps highlight the need for more comprehensive studies examining physical and psychological outcomes in diverse populations undergoing LSG during adolescence.

This study sought to assess the seven-years results of LSG in adolescent patients, focusing on weight loss efficacy and comorbidity resolution.

## Material and Methods

This retrospective investigation analyzed data from 63 adolescents, ranging in age from 10 to 19 years, who received LSG for morbid obesity in the form of antral resection [the first staple firing started 2 cm from the gastro duodenal junction (pyloric ring)] using 36-French bougie [13], at Tanta University Hospitals and affiliated hospitals, Egypt, between January 2014 and December 2015. Individuals who underwent alternative bariatric procedures, including Roux-en-Y gastric bypass and laparoscopic adjustable gastric banding, within the specified timeframe were not included in the study. The study design was reviewed and approved by both the institutional and regional ethical review boards (approval code: 36264PR194/5/23), and written informed consent was secured from all participants or their legal guardians before enrollment.

Medical records and electronic databases were reviewed to evaluate LSG outcomes over a 7-year follow-up period. All enrolled patients had previously undergone unsuccessful medical weight management trials for at least six months and received dedicated bariatric physician follow-up. Given the impact of obesity on adolescent well-being, preoperative assessments were conducted with both patients and guardians present.

Data collection encompassed demographic characteristics, physiological parameters, baseline preoperative weight was measured on the morning of surgery, comorbidities, the length of hospital stay, surgical operative time, and pre- and postoperative glycosylated hemoglobin (HbA1c) levels. BMI, percent excess weight loss (%EWL) = [(Initial weight – Postop weight)/(Initial weight—Ideal weight)] * 100, where ideal weight was deteremined based on a BMI of 25 kg/m^2^, percent total weight loss (%TWL) = [(Initial Weight—postop Weight)/Initial Weight] * 100, percent EBMI loss (%EBMIL) = [(Initial BMI – Postop BMI)/(Initial BMI – 25)] * 100 [14], and gastric volumetry by 3D Computed Tomography (3D-CT Volumetry) were assessed at baseline, 1, 3, 5, and 7 years postoperatively. Postoperative complications were identified, such as leaks, bleeding, and wound infection.

The study evaluated the resolution of several obesity-related comorbidities, including dyslipidemia, T2DM, obstructive sleep apnea, gastroesophageal reflux disease, and hypertension. Complete remission of T2DM was defined as the maintenance of normalized glucose metabolism (characterized by normal HbA1c levels and a fasting glucose level below 100 mg/dL) for a minimum of one year without the use of any pharmacological agents [15]. The resolution of hypertension was identified by the maintenance of normal blood pressure (systolic blood pressure less than 140 mmHg and diastolic blood pressure less than 80 mmHg) without the need for antihypertensive medications [16].

### Sample Size Calculation

The sample size was established using G*Power 3.1.9.2 software (Universität Kiel, Germany). A pilot study, comprising five cases demonstrated a mean (± SD) preoperative weight of 119 ± 25.3 kg and a one-year postoperative weight of 94.6 ± 14.7 kg. Based on these findings, the sample size was calculated with an effect size of 1.17, a 95% confidence level, and 95% statistical power, resulting in a minimum recruitment target of 40 individuals.

### Statistical Analysis

SPSS v26 (IBM^Inc.^, Chicago, IL, USA) was utilized for data analysis. Quantitative variables (Weight, BMI, gastric volumetry, %TWL, %EWL, %BMIL, waist circumference, thigh circumference, arm circumference and HbA1c) were presented as mean and standard deviation (SD) and compared between the measurements utilizing repeated measure ANOVA test. Qualitative variables (DM, hypertension, OSA, GERD and dyslipidemia) were presented as frequency and percentage and analyzed using the McNemar test. A two-tailed P value ≤ 0.05 was considered statistically significant.

## Results

**Fig. 1:**
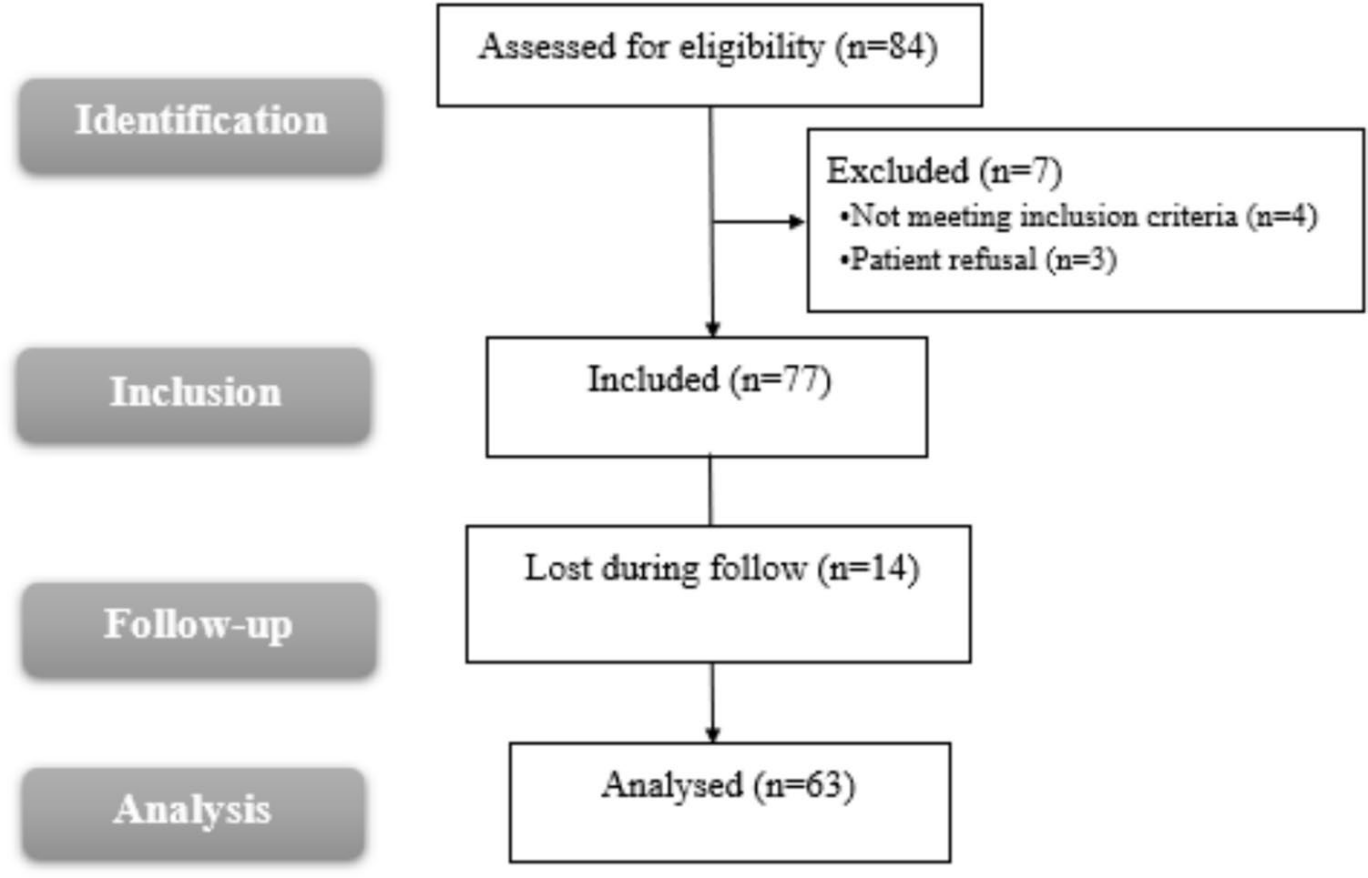
Flow chart of the studied patients

The enrollment process began with 84 patients who were assessed for eligibility; of these, four were excluded based on study criteria, and three refused participation. After accounting for fourteen patients lost to follow-up, statistical analysis was conducted on a final sample of 63 participants. Figure 1

The mean age of patients was 15.4 ± 2.39 years. The gender distribution showed a female predominance, with 39 patients (61.9%) compared to 24 male patients (38.1%). The surgical intervention had a mean operative duration of 74.6 ± 9.17 min, and patients typically remained hospitalized for 1.6 ± 0.5 days. Five patients (7.9%) experienced complications: 1 leak (1.59%), 1 bleeding (1.59%), and 3 wound infections (4.76%), with no overlap: the leak occurred in 1 (1.59%) patient from a staple line at the upper end; the patient was treated by mega stent (Niti-S Esophageal covered stent, TaeWoong Medical Co., Ltd., South Korea) through upper gastrointestinal endoscopy, that removed after 6 weeks after upper GI contrast to make sure no more leak, then removed by the endoscope. Bleeding occurred in another patient (1.59%) who was treated conservatively in the ICU, and there was no need for surgical intervention. Wound infection occurred in 3 (4.76%) patients at the port of extraction of the resected part of the stomach and was treated by antibiotics and good dressings. Table 1

**Table 1:** Demographic data, vital signs, operative time, hospital stay, and immediate postoperative complications of the studied patients

			(*n* = 63)
Age (years)			15.4 ± 2.39
Sex		Male	24 (38.1%)
		Female	39 (61.9%)
Operative time (min)			74.6 ± 9.17
Hospital stay (days)			1.6 ± 0.5
Complications	5 (7.94%)		
	Leak		1 (1.59%)
	Bleeding		1 (1.59%)
	Wound infection		3 (4.76%)

Data are presented as mean ± SD or frequency (%)

**Fig. 2:**
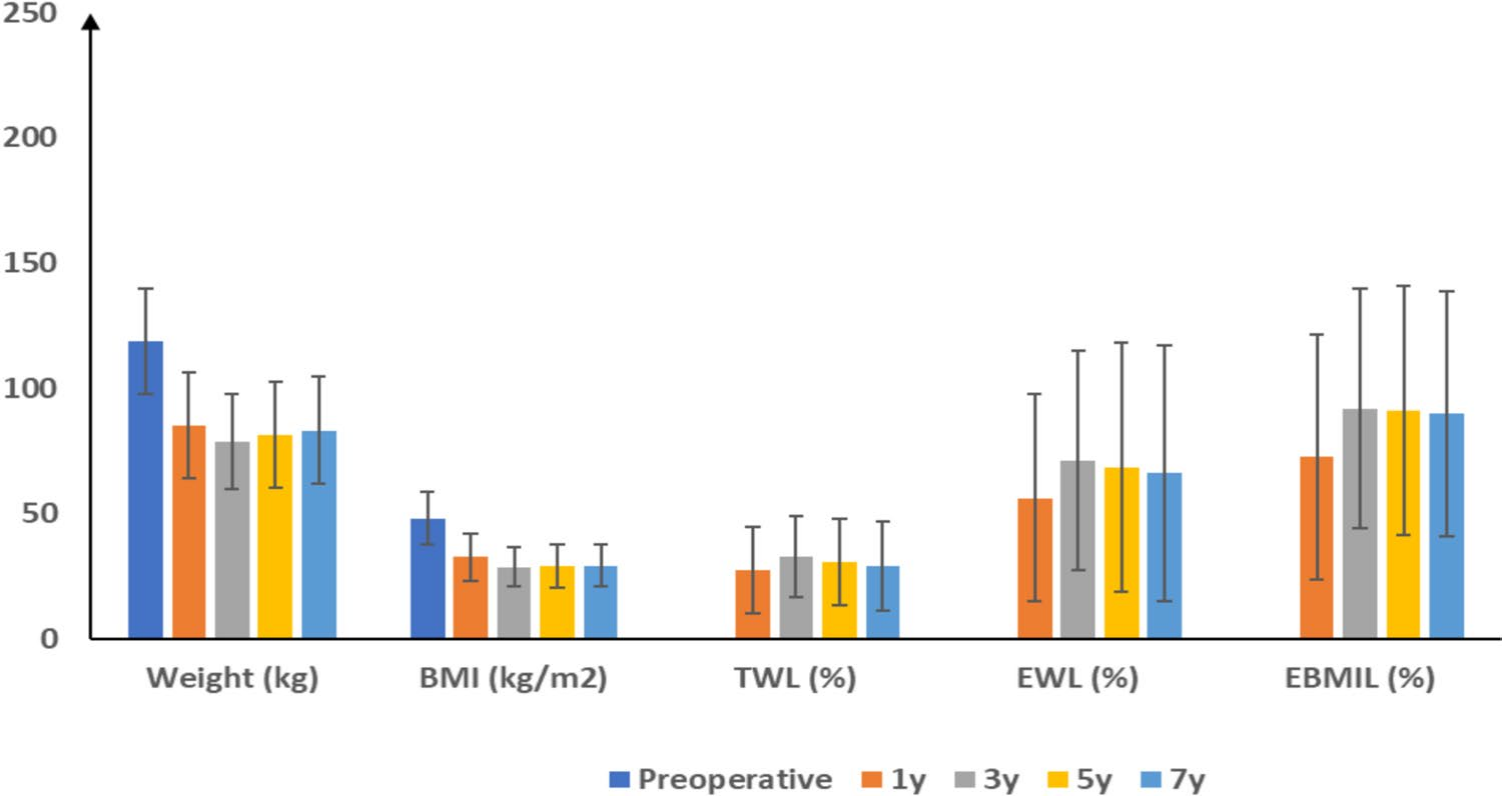
Weight, BMI, %TWL, %EWL, and %EBMIL measurements of the studied patients

There was a significant reduction across all measured parameters throughout the seven-years follow-up period. Mean body weight decreased from 118.8 ± 20.98 to 83.3 ± 21.49 kg in the 7 th year. From the lowest weight recorded at 3 years (78.7 ± 18.83 kg), patients regained a mean of 4.63 ± 5.18 kg by year 7 (p < 0.001). BMI substantially reduced from 48.1 ± 10.55 to 29.3 ± 8.53 kg/m.^2^. Percent TWL, EWL, and EBMIL significantly improved compared to the first postoperative year (p < 0.001). Percent TWL increased from 27.5 ± 17.26% at one year to peak at 32.9 ± 16.09% at three years before slightly declining to 29.1 ± 17.66% at seven years. Similarly, percent EWL improved from 56.3 ± 41.36% at one year to maximize at 71.3 ± 43.87% at three years, subsequently moderating to 66.1 ± 51.28% at seven years. Percent EBMIL increased from 72.6 ± 48.92% at one year to a peak at 92 ± 47.7% at three years before slightly declining to 90 ± 48.93% at seven years. Table 2, Fig. 2

**Table 2:** Weight, BMI, %TWL, %EWL, %EBMIL, and gastric volumetry measurements of the studied patients

	Preoperative	1 yr	3 yrs	5 yrs	7 yrs
Weight (kg)	118.8 ± 20.98	85.2 ± 21.27	78.7 ± 18.83	81.5 ± 20.97	83.3 ± 21.49
*P* value		< 0.001	< 0.001	< 0.001	< 0.001
BMI (kg/m^2^)	48.1 ± 10.55	32.7 ± 9.44	28.7 ± 7.9	29.1 ± 8.52	29.3 ± 8.53
*P* value		< 0.001	< 0.001	< 0.001	< 0.001
Gastric volumetry (ml)	1244.3 ± 251.39	181.4 ± 25.01	207.6 ± 25.38	258.3 ± 26	311 ± 29.93
*P* value		< 0.001	< 0.001	< 0.001	< 0.001
TWL (%)		27.5 ± 17.26	32.9 ± 16.09	30.6 ± 17.33	29.1 ± 17.66
*P* value			< 0.001	< 0.001	< 0.001
EWL (%)		56.3 ± 41.36	71.3 ± 43.87	68.6 ± 49.54	66.1 ± 51.28
*P* value			< 0.001	< 0.001	< 0.001
EBMIL (%)		72.6 ± 48.92	92 ± 47.7	91.1 ± 49.7	90 ± 48.93
*P* value			< 0.001	< 0.001	< 0.001

Data are presented as mean ± SD. *TWL* Total weight loss, *EWL* Excess weight loss, *EBMIL* Excess body mass index loss

Gastric volumetry markedly reduced from 1244.3 ± 251.39 ml preoperatively to 311 ± 29.93 ml at 7 years (p < 0.001). Table 2

Waist circumference, Thigh circumference, and Arm circumference were significantly lower at 1 yr, 3 yrs, 5 yrs, and 7 yrs compared to preoperative (P < 0.001). Table 3

**Table 3:** Anthropometric measurements of the studied patients

	Preoperative	1 yr	3 yrs	5 yrs	7 yrs
Waist circumference (cm)	105.5 ± 5.4	71.1 ± 7.67	60.2 ± 8	63.7 ± 8.25	65.7 ± 8.48
*P* value		< 0.001	< 0.001	< 0.001	< 0.001
Thigh circumference (cm)	66 ± 4.86	47.7 ± 6.36	42.5 ± 6.2	44.5 ± 6.39	46.5 ± 6.56
*P* value		< 0.001	< 0.001	< 0.001	< 0.001
Arm circumference (cm)	39.2 ± 4.36	24.7 ± 5.69	21.3 ± 5.66	22.8 ± 5.59	24.7 ± 5.68
*P* value		< 0.001	< 0.001	< 0.001	< 0.001

Data are presented as mean ± SD

Diabetes mellitus (DM) remission, number of patients decreased from 22 (34.92%) to 7 (11.11%) patients, and HbA1c levels improved from 6.4 ± 0.69 to 5.7 ± 0.77% (all p < 0.001). Table 4

**Table 4:** DM and HbA1c of the studied patients

	1 yr	3 yrs	5 yrs	7 yrs
DM	22 (34.92%)	10 (15.87%)	8 (12.7%)	7 (11.11%)
*P* value		< 0.001	< 0.001	< 0.001
HbA1c (%)	6.4 ± 0.69	6 ± 0.77	5.9 ± 0.77	5.7 ± 0.77
*P* value		< 0.001	< 0.001	< 0.001

Data are presented as mean ± SD. *DM* Diabetes mellitus

Also, other comorbidities like hypertension remission, number of patients decreased from 38 (60.32%) to 8 (12.7%) patients, and OSA from 39.68% to 6.35%. GERD prevalence declined from 25.4% to 4.76% at 7 years, with all persistent cases (n = 3) requiring revisional surgery. Dyslipidemia displayed the most dramatic improvement, declining from 42.86% to 3.17% (all p < 0.05). Table 5

**Table 5:** Comorbidities of the studied patients

	Preoperative	1 yr	3 yrs	5 yrs	7 yrs
Hypertension	38 (60.32%)	17 (26.98%)	14 (22.22%)	11 (17.46%)	8 (12.7%)
*P* value		< 0.001	< 0.001	< 0.001	< 0.001
OSA	25 (39.68%)	14 (22.22%)	12 (19.05%)	7 (11.11%)	4 (6.35%)
*P* value		< 0.001	< 0.001	< 0.001	< 0.001
GERD	16 (25.4%)	8 (12.7%)	5 (7.94%)	4 (6.35%)	3 (4.76%)
*P* value		0.008	< 0.001	< 0.001	< 0.001
Dyslipidemia	27 (42.86%)	12 (19.05%)	8 (12.7%)	4 (6.35%)	2 (3.17%)
*P* value		< 0.001	< 0.001	< 0.001	< 0.001

Data are presented as frequency (%). *OSA* Obstructive sleep apnea, *GERD* Gastroesophageal reflux disease

At the seven-year follow-up, most patients (79.37%) remained single, while 13 (20.63%) married. Fertility outcomes were favorable, with only one patient (1.59%) reporting fertility issues. Among the 20.6% (n = 13) who married by follow-up, 76.9% (n = 10) achieved successful pregnancies. Given the small subgroup (n = 13), these findings require cautious interpretation. Table 6

**Table 6:** Marital status, infertility, and complete successful pregnancy of the studied patients

		(*n* = 63)
Marital status	Single	50 (79.37%)
	Married	13 (20.63%)
Infertility		1 (1.59%)
Complete successful pregnancy		10/13 (76.92%)

Data are presented as frequency (%)

## Revisional Surgery

One patient (1.59%) had repeated vomiting with nutritional deficiencies for about 18 months. 3D volumetry revealed stricture at the incisura, and a laparoscopic gastric bypass was done, and doing well after. Three patients (4.76%) had intractable GERD that did not respond to medical treatment, 2 had laparoscopic gastric bypass, and the third refused bypass, asked for cruroplasty with medical treatment, and she is doing well. Two patients (3.17%) lost less than 50% of excess weight after 2 years post sleeve despite adherence to lifestyle modifications, strict dietary habits, and no technical problems as proved by volumetry; asked for gastric bypass, in one weight loss is improved, but the other is not what she hoped, and no cause could be detected for this. Three patients (4.76%) experienced weight regain after 5 years from sleeve gastrectomy, accompanied by gastric dilatation observed on volumetry, along with poor dietary habits, and lack of adherence to recommended nutritional guidelines. While an increase in gastric volume was documented, causality between volumetric changes and weight regain cannot be definitively established due to concurrent behavioral factors. One underwent revisional sleeve gastrectomy, due to generalized gastric tube dilatation, despite the controversial nature of this approach. The decision was made after multidisciplinary evaluation, including endoscopic and radiological findings, and patient preference. The other 2 underwent laparoscopic gastric bypass. Table 7

**Table 7:** Redo surgery of the studied patients

	(*n* = 63)
Repeated vomiting	1 (1.59%)
Intractable GERD	3 (4.76%)
Loss less than 50% of excess weight after 2 years	2 (3.17%)
Weight regain after 5 years	3 (4.76%)

Data are presented as frequency (%)

## Discussion

Laparoscopic sleeve gastrectomy has become a viable intervention for treating severe obesity in adolescents, with mounting evidence supporting its long-term efficacy and safety [7, 8]. The growing prevalence of adolescent obesity and its associated comorbidities has led to increased interest in surgical interventions, mainly as conservative treatments often yield insufficient results in this population [17, 18].

Our cohort comprised 63 adolescents with a mean age of 15.4 ± 2.39 years, showing female predominance (61.9%). This gender distribution aligns with several contemporary studies, including Al Sabah et al. [3], who reported 71% female patients, and Alqahtani et al. [7], with 55% female representation. Our study’s mean operative duration of 74.6 ± 9.17 min was comparable to Berry et al. [19], who reported a median time of 86.9 min. Our complication rates were notably low, with leak and bleeding rates of 1.59% each, like Başalan et al. [20], who reported 1.6% for both complications. Our study’s wound infection rate of 4.76% was slightly higher than typically reported in the literature, suggesting the need for enhanced perioperative infection control protocols.

To contextualize our findings, we compared our seven-year outcomes with those reported in international literature on adolescent LSG. Table 8 summarizes key parameters including weight loss, comorbidity resolution, and revision rates, highlighting the consistency of our results with established evidence.

**Table 8:** Comparison of 7-Year LSG outcomes in adolescents with international literature

Parameter	Your study (7 yrs) (n = 63)	Comparable studies	Sample size	Interpretation
BMI Reduction	48.1 → 29.3 kg/m^2^	Alqahtani et al. (2021) [7]: 45.7 → 30.2 kg/m^2^ (10 yrs) Goldenshluger et al. (2023) [18]: 44.9 → 30.1 kg/m^2^ (10 yrs)	108 52	Similar long-term BMI reduction trend
%TWL (Total Weight Loss)	Peaked at 32.9% (3 yrs), declined to 29.1% (7 yrs)	de la Cruz-Muñoz et al. (2022) [8]: ~ 31.3% at long-term	75	Consistent weight loss profile
DM Remission (T2DM)	34.9% → 11.1%; HbA1c: 6.4% → 5.7%	Mostafa et al. (2022) [21]: 84.3% remission at 1 yr Schauer et al. (2017) [22]: sustained remission over 5 yrs	30 150 (adults)	Comparable remission; strong metabolic control
Hypertension Remission	60.3% → 12.7%	Ruiz-Cota et al. (2019) [23]: ~ 75% remission Salminen et al. (2022) [24]: sustained improvement	1200 (meta-analysis) 240 (adults)	Superior resolution rate
Dyslipidemia Resolution	42.9% → 3.2%	Ruiz-Cota et al. (2019) [23]: 75% remission Sjöström et al. (2007) [25]: improved lipid profile	1200 (meta-analysis) 4047 (adults)	Stronger remission than typical
OSA (Sleep Apnea) Improvement	39.7% → 6.4%	El-Matbouly et al. (2018) [26]: 64% cure rate Greenburg et al. (2009) [27]: significant OSA relief	50 342 (meta-analysis)	Aligned with international outcomes
GERD Prevalence	25.4% → 4.8%	Noel et al. (2017) [28]: GERD increased to 31% (adults) Braghetto et al. (2010) [29], Daes et al. (2012) [30]: GERD improved with surgical technique	126 30	Better than adult outcomes; likely due to patient age and technique
Revisional Surgery Rate	~ 14% (varied causes)	Fischer et al. (2012) [31]: 6.8% Ben-Porat et al. (2021) [32]: 10–15% over 8 years	1200 (meta-analysis) 212	Within expected range
Gastric Volumetry	1244 ml → 311 ml	Pañella et al. (2020) [33]: 216.7 → 367.5 ml (1–5 yrs)	45	similar reduction; volume changes not directly predictive of weight
Fertility Outcomes	76.9% successful pregnancies among married (n = 13)	İlyas et al. (2023) [34]: 91.3% conception Musella et al. (2012) [35]: 62.7% pregnancy rate	80 110	Favorable, but small adolescent cohort limits generalizability

The substantial reduction in mean body weight from 118.8 ± 20.98 to 83.3 ± 21.49 kg at seven years demonstrates the procedure's long-term effectiveness. Our BMI reduction from 48.1 ± 10.55 to 29.3 ± 8.53 kg/m^2^ is particularly noteworthy compared to Goldenshluger et al. [18], who reported a decrease from 44.94 to 30.11 kg/m^2^ at 10 years. The modest weight regain of 4.63 ± 5.18 kg observed in our study aligns with Yang et al. [36], who found a 20.1% weight regain rate at three years. Our percent TWL peaked at 32.9% at three years, comparable to de la Cruz-Muñoz et al. [8], who reported 31.3% TWL at long-term follow-up.

The reduction in DM prevalence from 34.92% to 11.11% over seven years represents a significant achievement in metabolic improvement. This outcome compares favorably with Mostafa et al. [21], who reported an 84.3% diabetes remission rate at one year. Also consistent with the long-term outcomes reported by Schauer et al. [22], who observed durable remission of type 2 diabetes following bariatric surgery compared to intensive medical therapy. The improvement in HbA1c levels from 6.4 ± 0.69% to 5.7 ± 0.77% demonstrates effective glycemic control, aligning with Elhag and Ansari [17] findings of sustained metabolic improvements up to 9 years post-surgery. Similarly, our HbA1c improvements align with the metabolic benefits reported by Mingrone et al. [37], who showed significant glycemic control up to five years postoperatively.

The marked reduction in hypertension rates from 60.32% to 12.7% exceeds the outcomes reported by Salminen et al. [24] in their 10-year follow-up study, and supports the cardiovascular benefits of LSG. In parallel, the marked improvement in dyslipidemia (from 42.86% to 3.17%) mirrors the lipid profile improvements seen in the Swedish Obese Subjects study by Sjöström et al. [25], surpasses the outcomes reported by Ruiz-Cota et al. [23], who noted a 75% dyslipidemia remission rate in their systematic review, where bariatric surgery yielded sustained reductions in total cholesterol and triglycerides.

The improvement in OSA from 39.68% to 6.35% is consistent with El-Matbouly et al. [26], who reported a 64% cure rate for OSA. Furthermore, our observed reduction in obstructive sleep apnea (OSA) corresponds with findings by Greenburg et al. [27], who documented significant improvement in OSA severity following weight loss from bariatric procedures. These comparisons further reinforce the effectiveness of LSG in resolving obesity-related comorbidities during adolescence.

Gastroesophageal reflux disease (GERD) after sleeve gastrectomy remains a controversial topic in bariatric literature. While LSG is widely accepted for its metabolic benefits and relative technical simplicity, it has been associated with both improvement and new onset of GERD. In our study, GERD prevalence decreased significantly from 25.4% preoperatively to 4.76% at seven years postoperatively. This finding contrasts with some adult studies that have reported increased GERD incidence following LSG. For example, Noel et al. [28] reported a GERD rate of 31% at 8 years postoperatively, attributing the increase to anatomical changes such as reduced gastric compliance, increased intragastric pressure, and disruption of the angle of His.

Several factors may account for the favorable GERD outcomes in our adolescent cohort. First, younger patients may have more adaptable esophageal motility and less baseline esophagitis, leading to better long-term adaptation. Second, our surgical technique involved initiating the staple line 2 cm from the pylorus and using a 36-French bougie, which may increase the gastric emptying and reduce reflux-promoting mechanisms such as excessive intragastric pressure or sleeve narrowing. Additionally, we ensured careful attention to avoid twisting, kinking, or narrowing at the incisura angularis, which are technical causes of postoperative reflux.

Our findings are more consistent with studies such as those by Braghetto et al. [29], and Daes et al. [30], who demonstrated that GERD symptoms may improve postoperatively when surgical technique is optimized and anatomical factors such as hiatal hernia are addressed intraoperatively. Furthermore, a study by Howard et al. [38], found that younger age and shorter reflux history are predictive of better postoperative GERD outcomes.

However, the long-term impact of LSG on GERD remains nuanced. A subset of patients in our cohort (4.76%) required further surgical intervention due to intractable reflux, including conversion to Roux-en-Y gastric bypass or cruroplasty. This highlights that while LSG may be associated with GERD resolution in many cases, it can also lead to refractory symptoms in others, necessitating individualized risk assessment and long-term follow-up.

These findings support the need for preoperative GERD evaluation and tailored surgical planning. Future prospective studies with pH monitoring, manometry, and endoscopic follow-up would be valuable in clarifying the mechanisms and long-term trajectory of GERD in adolescent LSG patients.

Our study’s revision rate was notable, with various indications including stricture (1.59%), intractable GERD (4.76%), and insufficient weight loss (3.17%). These rates are comparable to Fischer et al. [31] systematic review, which reported reoperation rates of 6.8%. The weight regain-related revisions in our study (4.76%) align with Ben-Porat et al. [32] findings, where higher baseline BMI and younger age were identified as predictors of increased weight regain.

The significant reduction in gastric volumetry from 1244.3 ± 251.39 to 311 ± 29.93 ml observed in our study warrants particular attention. This finding can be contextualized with Pañella et al. [33] research, which demonstrated increased gastric reservoir volume from 216.7 mL at 1 year to 367.5 mL at 5 years post-surgery. Our observed gastric volume reduction (75% from baseline) exceeded Pañella et al.’s findings (367.5 mL at 5 years), possibly due to our consistent antral resection technique and younger patient cohort. While volume expansion occurred in patients with weight regain, behavioral factors (e.g., dietary non-adherence) confound causality, underscoring the need for multimodal follow-up. The difference in final volumes between studies suggests that maintaining a smaller gastric volume may contribute to superior weight loss outcomes. However, the association involving gastric volume and weight loss outcomes continues to be complex, as Pañella et al. [33] found that the association concerning volume and weight loss parameters dissipated by the 5-year mark.

Our study provided valuable data on social and fertility outcomes, showing that 20.63% of patients had married by the seven-year follow-up, with favorable fertility outcomes (76.92% successful pregnancies among married patients). These findings align with several studies in the literature examining fertility outcomes after bariatric surgery.

İlyas et al. [34] reported even higher conception rates in their cohort, with 91.3% of patients conceiving post-LSG. Their study demonstrated a significant correlation between BMI reduction and successful conception (p = 0.001), suggesting that weight loss improves fertility outcomes. Our outcomes matching Musella et al. [35] retrospective analysis of 110 obese infertile women, which identified post-surgical weight loss (OR 20.2, p = 0.001) and achieved BMI as significant predictors of successful conception. Their study reported successful pregnancies in 62.7% of participants, with all pregnancies resulting in live births without complications. Interestingly, Dilday et al. [39] informed that 22% of patients with polycystic ovary syndrome achieved pregnancy within 12 months after surgery, with 69% of these pregnancies occurring in women who had not previously given birth. These studies differ from ours in that they were working on adults; most of them are married or going to marriage and seeking to conceive, our populations mostly were young at time of surgery and not all of them get married at the 7 years follow-up.

Despite its strengths, our study has important limitations. The retrospective nature and single-institution setting may restrict generalizability. The sample size, while reasonable for long-term adolescent data, remains modest. We also did not incorporate standardized measures of psychosocial outcomes, such as quality of life or mental health assessments, which are crucial for evaluating the broader impact of bariatric surgery in adolescents**.**

## Conclusions

This seven-year follow-up study supports LSG as an effective long-term intervention for adolescents with severe obesity. The procedure resulted in sustained weight loss, significant reduction in BMI, and meaningful improvements in obesity-related comorbidities. While the majority of patients maintained positive outcomes, a subset experienced weight regain or required revisional surgery, underscoring the need for ongoing clinical follow-up and behavioral support. Preliminary data suggest favorable fertility outcomes, but larger studies are needed. These findings reinforce the role of LSG as a long-term treatment option in carefully selected adolescents but highlight the importance of individualized care, thorough preoperative evaluation, and acknowledgment of the procedure’s limitations in broader clinical applications.

## Data Availability

No datasets were generated or analysed during the current study.
